# Extraction Optimization, Functional and Thermal Properties of Protein from Cherimoya Seed as an Unexploited By-Product

**DOI:** 10.3390/foods11223694

**Published:** 2022-11-18

**Authors:** Jose C. Orellana-Palacios, Milad Hadidi, Marwa Yassamine Boudechiche, Maria Lopez S. Ortega, Diego J. Gonzalez-Serrano, Andres Moreno, Przemysław Łukasz Kowalczewski, Matteo Bordiga, Amin Mousavi Khanegah

**Affiliations:** 1 Department of Organic Chemistry, Faculty of Chemical Sciences and Technologies, University of Castilla-La Mancha, 13071 Ciudad Real, Spain; 2 Department of Food Technology of Plant Origin, Poznań University of Life Sciences, 31 Wojska Polskiego St., 60-624 Poznań, Poland; 3 Department of Pharmaceutical Sciences, Università Degli Studi del Piemonte Orientale “A. Avogadro”, Largo Donegani 2, 28100 Novara, Italy; 4 Department of Fruit and Vegetable Product Technology, Prof. Wacław Dąbrowski Institute of Agricultural and Food Biotechnology—State Research Institute, 36 Rakowiecka St., 02-532 Warsaw, Poland

**Keywords:** thermal stability, plant proteins, functionality, extraction, by-product

## Abstract

Plant-based proteins are gaining in attraction compared with animal-based proteins due to their superior ethical profiles, growing concerns on the part of various organizations about animal health and welfare, and increased global greenhouse-gas emissions in meat production. In this study, the response surface methodology (RSM) using a Box–Behnken design (BBD) was applied to optimize the ultrasound-assisted alkaline extraction of cherimoya-seed proteins as valuable by-products. The effects of three pH, temperature, and time factors on the protein-extraction yield and protein content were investigated. The pH at 10.5 and temperature of 41.8 °C for 26.1 min were considered the optimal ultrasound-assisted alkaline-extraction conditions since they provided the maximum extraction yield (17.3%) and protein content (65.6%). An established extraction technique was employed to enhance the cherimoya-seed protein yield, purity, and functional properties. A thermogravimetric analysis (TGA) of the samples showed that the ultrasound-assisted alkaline extraction improved the thermal stability of the protein concentrate.

## 1. Introduction

Cherimoya (*Annona cherimola*) is an edible fruit tree belonging to Annonaceae, commonly known as chirimoya, cultivated in subtropical or tropical areas. In the 17th century, the cherimoya was introduced to Spain from Latin America. Currently, Spain is the largest producer of cherimoya in the world [1]. Cherimoya is used extensively in local traditional medicine for its health-related beneficial activities against several digestive disorders and chronic diseases [2]. Beverages, jam, infusions, marmalades, and other traditional foods are made from the fruits and leaves of cherimoya. In several studies, bioactive compounds and phytochemicals isolated from plant organs have been demonstrated to have antimicrobial, antioxidant, antihyperglycemic, anti-inflammatory, and anticancer activities [3]. It contains characteristic secondary metabolites, such as saponins, coumarins, isoflavones, and alkaloids. Phenolic compounds, acetogenins, and terpenes are the main bioactive compounds in cherimoya seeds, leaves, and fruit, respectively [4]. Furthermore, cherimoya seeds as by-products could be valuable natural sources of protein to be employed as ingredients [5].

Today, plant-protein sources are gaining in attraction compared with meat proteins due to their superior ethical profiles, growing concerns on the part of organizations over animal health and welfare, and increased global greenhouse-gas emissions in animal-based products [6]. A few influential properties of plant proteins are considered in food products, such as amino-acid composition, secondary and tertiary structure, molecular weight, isoelectric point, net electrostatic charge, emulsifying, foaming, and water-oil absorbing capacities, as well as solubility [7]. Protein functionality, nutritional characteristics, and gastrointestinal availability are influenced by structural changes, particularly in relation to the solution’s environmental conditions, such as pH, temperature, and ionic strength [8,9]. Different extraction solvents (e.g., alkali, water, acids, and organic) are usually used in chemical-protein-extraction methods. The conventional alkaline-extraction-acidic-precipitation method is widely used to produce protein concentrate and isolates [10,11]. Several emerging technologies have been used to enhance the extraction yield and reduce the duration and alkali consumption through alkaline extraction. Notably, assistance technologies are necessary for isolating intracellular proteins from the non-starch polysaccharides of the plant-cell wall. When used to disrupt cell walls, some innovative technologies, such as ultrasound, can enhance the extraction yield and functionality of plant-based proteins [12,13]. Ultrasound technology has been the subject of research as a green/eco-friendly method. Ultrasound-assisted extraction (UAE) is simple and inexpensive, can be applied to industrial and commercial production on a large scale, and can enhance mass transfer, thereby shortening the time of extraction, reducing the solvent consumption and temperature while lowering energy input and offering environmental protection in comparison with conventional extraction methods [14]. Ultrasound technology is often referred to as an enzymatic or chemical approach to food processing because it has a proper modification effect on the protein structure and functionality. It is generally more economical and easier to adapt and use in industry [15].

To the best of our knowledge, there are no reports on the extraction optimization of protein from cherimoya seed by employing an ultrasound-assisted alkaline-extraction method. The evaluation of the functional and thermal properties of cherimoya-seed-protein concentrate obtained by the ultrasound-assisted alkaline-extraction method has not yet been reported. In order to achieve a higher protein-extraction yield and protein content, an ultrasound-assisted alkaline-extraction technique was employed to extract protein concentrate from cherimoya seed and optimized by the Box–Behnken design (BBD) of response surface methodology (RSM). The extraction yields, purities, thermal and techno-functional properties such as water-holding and oil-binding capacities, and foaming properties of this protein were measured and compared with the classic alkaline-extraction method.

## 2. Materials and Methods

### 2.1. Ultrasound-Assisted Alkaline Extraction of Protein from Cherimoya Seeds

Ultrasound-assisted alkaline protein extraction was applied based on the method described by Hadidi et al. [16], with some modifications. In a beaker, the finely ground powder was dissolved in distilled water at three ratios of solvent/solid (10–30 mL/g). The pH of the mixture was adjusted to 9–11 with 1 M NaOH. Ultrasound treatment was accomplished using a 35-kilohertz ultrasonic cleaner bath (DT 1028 CH, Sonorex Digitec, Berlin, Germany) with a heater to set the required output power (300 W) in the range of 30–50 °C for 20–60 min. The ultrasonic output powers were measured calorimetrically, conforming to the method explained in Hadidi et al. [17]. The temperature T was recorded with a thermocouple as a function of time under adiabatic conditions. From temperature-versus-time data, the initial temperature rise dT/dt was determined by polynomial curve fitting. The absolute ultrasonic power P was calculated as

$$P={m\;c}_{p}\;\mathrm{dTdt}$$

where m is the total mass and c_p_ is the heat capacity of the solvent.

The obtained mixture was centrifuged at 7802× *g* for 20 min in a Medtronic BL-S centrifuge (7001448 head, J.P. Selecta, Madrid, Spain). The obtained extract of protein was acidified to pH 4 by 2 M HCl. Centrifugation was employed at 7802× *g* for 20 min for protein precipitation. The tubes were washed with distilled water to remove all soluble residuals, and the pH was adjusted at 7.0 by 1 M NaOH. The obtained retentate was dried with a freeze dryer (LioQuest-85/230 V-50 Hz, Telstar, Barcelona, Spain) at −80 °C for 24 h. The protein content was measured by the Kjeldahl method. Firstly, one gram of protein extracted was digested in 15 mL of concentrated sulfuric acid with a Kjeldahl catalyst (6.25% in CuSO_4_·5H_2_O, Merck, Darmstadt, Germany) in a heat-resistant tube at a temperature of ∼400 °C. After the digestion step, the cooled digest was diluted with distilled water. A total of 60 mL of NaOH (35%) was added to the solution and then subjected to steam distillation, which separated the volatile NH_3_ from the other constituents. The condensed NH_3_ was then trapped in dilute boric acid (1%). After titration by HCL (0.1 M), the nitrogen was measured, and the protein content was calculated by a conversion factor of 6.25 [18].

### 2.2. Conventional Alkaline Extraction

The experimental procedure for obtaining the proteins by the conventional method was carried out using only the optimal extraction conditions obtained from the results. This method was similar to that seen in [19], with minor changes. Briefly, a mixture of cherimoya-seed powder and distilled water was adjusted to pH 10.5 with 1 M NaOH, after which it was heated for 26 min at 42 °C and constantly stirred. The rest of the process was similar to that described in the ultrasound-assisted extraction. The protein content was measured by the Kjeldahl method.

### 2.3. Optimization of the Ultrasound-Assisted Alkaline-Extraction Process

Response surface methodology was used to optimize the process of protein extraction from cherimoya seeds. A three-factor Box–Behnken design with two responses was created for this task. The table resulting from the design corresponds to Table 1, which can be seen in the “Results and Discussion” section of this work. The optimization consisted in performing a certain number of experiments in which the value of each selected factor varied. In this way, a multitude of results was obtained for the desired responses and using the data, an analysis of variance (ANOVA) table capable of indicating whether the chosen factors used were significant for each response and whether the model created was adequate was created. In this work, the independent factors chosen were the solution’s pH, temperature, and extraction time (A: 9, 10, 11; B: 30, 40, 50 °C; and C: 20, 40, 60 min, respectively). The responses for these factors were extraction yield (%) and protein content (%). Perturbation plots were represented with the values obtained from the experiments to make the changes in the responses more visual according to the variations of each factor.

With the data from the ANOVA table, two second-degree polynomial equations were created with only the factors considered significant:

$$Y=\alpha_{m}+\alpha_{A}A+\alpha_{B}B+\alpha_{C}C+\alpha_{AB}AB+\alpha_{AC}AC+\alpha_{BC}+\alpha_{A^{2}}A^{2}+\alpha_{B^{2}}B^{2}+\alpha_{C^{2}}C^{2}$$

The characters in the equation that were not mentioned above, such as *α_m_*, *α_A_*, *α_B_*, represent the coefficients obtained in the ANOVA table for the model, for each factor, for the influence of two factors on a response, and the quadratic coefficients of each factor, and only appeared in the equation if they were factors with significance for the model. Three-dimensional response-surface plots were also created to make the influence of two factors on each response more visual, as with the perturbation plots.

### 2.4. Thermal Stability

The thermal stability of the proteins extracted by conventional and ultrasound-assisted methods was determined by thermogravimetric analysis (TGA 550, TA Instruments, New Castle, DE, USA). This method is similar to that used by [19], with some changes. In summary, 10 mg of protein extract were weighed in a platinum pan and subjected to the following conditions: argon flow (50 mL/min) and heating range of 10 °C/min up to 900 °C.

### 2.5. Techno-Functional Properties

#### 2.5.1. Water-Holding Capacity

The method used to measure this property was that employed by Hadidi et al. [19]. A mixture of 20 mL of distilled water and 1 g of protein was left for 80 min and centrifuged. The resulting volume of water was measured. The holding capacity was calculated by subtracting the water’s initial weight from the water’s final weight and dividing it by the grams of protein used (g water/g protein).

#### 2.5.2. Oil-Binding Capacity

The method described by Hadidi et al. [19], with minor modifications, was taken for the water-holding-capacity determination. In brief, the mixture of 1 g of protein and 10 g of oil was allowed to stand for 30 min. Next, centrifugation was carried out, and the free oil was measured. The binding capacity was calculated by subtracting the initial weight of oil minus the final weight of oil and dividing it by the grams of protein used (g oil/g protein).

#### 2.5.3. Foaming Capacity and Stability

These two properties were calculated following the method seen in [17]. Using a homogenizer (OV-5, Labolan, Navarra, Spain), 10 mL of 0.5% (*w*/*v*) protein solution was shaken for 2 min. The exact volumes were measured before and after shaking, and then the volume was measured again after 30 min of waiting at the same temperature. The percentage values for each property were calculated as follows: for the foaming capacity, the volume present after agitation was subtracted from the volume before agitation, and the result was divided by the latter volume. Subsequently, the result was multiplied by 100. In the case of foaming stability, it was undertaken by subtracting the volume after 30 min minus the volume after finishing the agitation and dividing by the latter again. Subsequently, the result was multiplied by 100.

## 3. Results and Discussion

### 3.1. Model Fitting and Statistical Analysis

When the ultrasound technique is used to obtain protein from vegetable matter, several factors are directly related to the extraction yield of the process and the protein content obtained in the extracts, such as extraction time, temperature, and pH of the mixture to be sonicated. In this experiment, 16 runs were carried out, and the three conditions were changed to determine how both the extraction yield and the protein content varied. The results of the experiments can be seen in Table 1. They were obtained using a Box–Behnken design for three factors with two responses.

According to the data concerning the extraction yield, significant changes were observed in the values obtained, ranging from 9.1 to 18.7%. The changes in the protein content obtained in the extracts were more pronounced, ranging from 28.3 to 67.4%. These results demonstrate that variations of the ultrasound-mediated extraction-process factors greatly influenced the outcome of the process.

Another source of information from which to draw other results is the ANOVA table (Table 2). It shows both coefficients that indicate whether the proposed models are adequate, whether the results obtained are in line with those predicted by the software, and information about the significance of the parameters used. The correlation coefficient (R^2^) was the parameter used to determine the adequacy of the models. As can be seen, the R^2^ value for both responses is close to 1 (0.9680 for the extraction yield and 0.9737 for the protein content), which means that 96% and 97% of the variations obtained could be explained for both of them. The coefficient of variation (CV) and the adj-R^2^ coefficient of the models can also be observed in Table 2, which indicates the similarity between the values predicted and those obtained. The CV percentage was below 10% in both responses (6.06 and 7.51); therefore, it can be stated that the values obtained were in accordance with the model. In addition, the adj-R^2^ coefficient values for both responses were sufficiently high (0.9299 and 0.9344) to confirm this last statement. Furthermore, the significance of the two models was checked by examining their *p*-factor and F-factor values. For the extraction yield, the model calculated 0.0008 and 20.15 for the *p*-value and F-value, respectively. For the protein content, the values were 0.0005 and 24.72, respectively. The low values of the p-factor and the high values of the F-factor confirmed the significance of the two models [17]. With all this information, it is possible to affirm that the models for the optimization of the ultrasound-assisted protein-extraction process that was used, and all their results, have a guarantee of quality since several statistical factors were used to verify each model created.

### 3.2. Influence of Process Variables on Responses

Given the small amount of protein generally present in the seeds of various fruits, a rather low extraction yield was expected from the cherimoya seeds’ extraction process [20]. As can be seen in Table 1, the highest extraction-yield value of the process was 18.7%, corresponding to the following extraction conditions: pH 10, 50 °C, and 20 min of extraction. Therefore, it was concluded that these conditions were the best as long as a higher extraction yield of the process was sought without considering other parameters (such as protein content).

In order to examine the influence of each variable independently concerning the extraction yield, a second-degree polynomial equation was created using the data from Table 2, where the *A*, *B*, and *C* coefficients are pH, temperature, and time, respectively:

$$Yex.y=\;+\;15+2.26A+1.21B-2.13C-1.80BC-1.19A^{2}-1.24B^{2}$$

It can be seen how the coefficients shown (*A*, *B*, and *C*) all had a significant influence on the performance of the extraction process (*p* < 0.05 in the three of them). Another glance at the table shows that the interactions between pH and temperature and those between pH and time did not influence the protein-extraction yield. The same was true for the quadratic regression coefficient of the time parameter (*p* > 0.05 in all of them). The interactions between temperature and time and the quadratic regression of pH and temperature all had an influence (*p* < 0.05 in the three of them) on the extraction yield [21].

Turning now to the protein-content results, the most favorable conditions for achieving the highest percentage of protein in the extracts obtained were pH 10, 40 °C, and 40 min of extraction, according to the results shown in Table 1.

Again, a second-degree polynomial equation was created with the data in Table 2 to observe the influence of each parameter individually concerning the protein content obtained:

$$Ypr.c=\;+\;65.98+5.61A-4.39B-7.05C-5.62AB-5.25AC-5.50BC-\;11.72A^{2}-12.27B^{2}-12C^{2}$$

All the three main coefficients (*A*, *B*, and *C*) had a significant influence on the protein content of the extracts (*p* < 0.05). In this case, the interactions between pH and temperature, pH and time, and temperature and time influenced the protein content obtained. The same was true for the quadratic regression coefficients (*p* < 0.05 in all).

**Figure 1:**
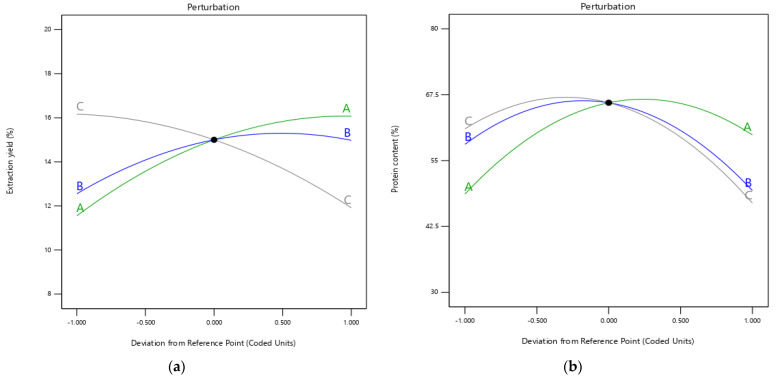
Perturbation plots representing the effect of extraction factors (A: pH; B: temperature; C: extraction time) on extraction yield (**a**) and protein content (**b**) of cherimoya-seed protein concentrate.

Another way to show the influence of the three parameters affecting the extraction yield and the amount of protein obtained is through perturbation plots. These are displayed in Figure 1, which shows how the values of the extraction yield and protein content increased or decreased as a function of how the parameters used changed. Figure 1a shows the increasing trend of the extraction yield as the pH increased to about 10.5; later, it demonstrated a slightly reducing trend. This phenomenon could be explained by the protein denaturation at high pH during harsh alkaline conditions [22]. Similar to the pH effect, an increase in extraction yield was observed when the temperature was raised from 30 to 40 and then to 50 °C, reaching above 14% of the extraction yield. The higher temperatures caused a change in the trend of the extraction yield, indicating that a further increase in temperature would negatively affect the extraction yield and that it is better to work at low temperatures, as has been seen in other cases [23]. In the case of the extraction time, a decrease in extraction yield was observed at longer times, to below 12%. Previously published studies have shown that excessive extraction time is counterproductive in obtaining a good protein-extraction yield [17,24].

In Figure 1b, the results were different. On the one hand, an increase in pH from 9 to 10 favored obtaining a higher protein content, and if this factor was increased more, a maximum protein-content value slightly lower than 67.5% was reached. However, when the pH was raised to 11, there was a decrease in the protein content obtained; the final value was around 60%. An explanation for this is that, as in extraction yield, more aggressive pH conditions lead more easily to protein denaturation. On the other hand, it can be seen how a slight increase in temperature can favor higher protein content. This is because better mass transfer occurs when the temperature is partially increased so that proteins can pass into the medium more easily [25]. However, when 40 °C was reached, this effect no longer occurred; from that point on, lower amounts of protein were obtained. The lowest value was obtained when the temperature reached 50 °C (52%). This is because high temperatures can cause protein denaturation. Temperature and time had comparable effects. Both curves were very similar, increasing slightly in the first section but beginning to decrease before reaching the value of the central point. From this point, a sharp drop occurred until 60 min of extraction time, at which point a protein percentage of less than 50% was obtained. This is because the ultrasound technique can modify the properties of proteins. When the extraction time is too long, proteins can undergo changes in their structure that cause them to aggregate [26].

**Figure 2:**
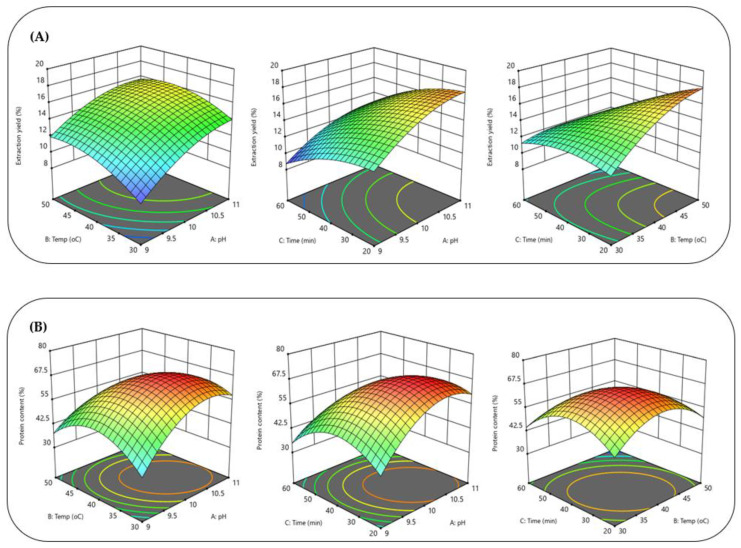
Response surface plots representing the effect of ultrasound-assisted alkaline-extraction conditions on the (**A**) extraction yield and (**B**) protein content of cherimoya-seed protein concentrate.

Another method of analyzing the data obtained from the experiments was the use of three-dimensional response surface plots, through which the influence of two variables simultaneously on the extraction yield or the extracted protein content was observed. Figure 2 shows a three-dimensional graphical representation of this. In the case of the extraction yield, although neither the interactions between pH and temperature (A and B) nor the interactions between pH and time (A and C) were significant, the situation was different between the temperature and the extraction time (B and C). The interactions between the pH and the temperature (A and B), the pH and the time (B and C), and the temperature and the time (B and C) all influenced the protein content obtained [17].

### 3.3. Optimized Extraction Conditions

**Figure 3:**
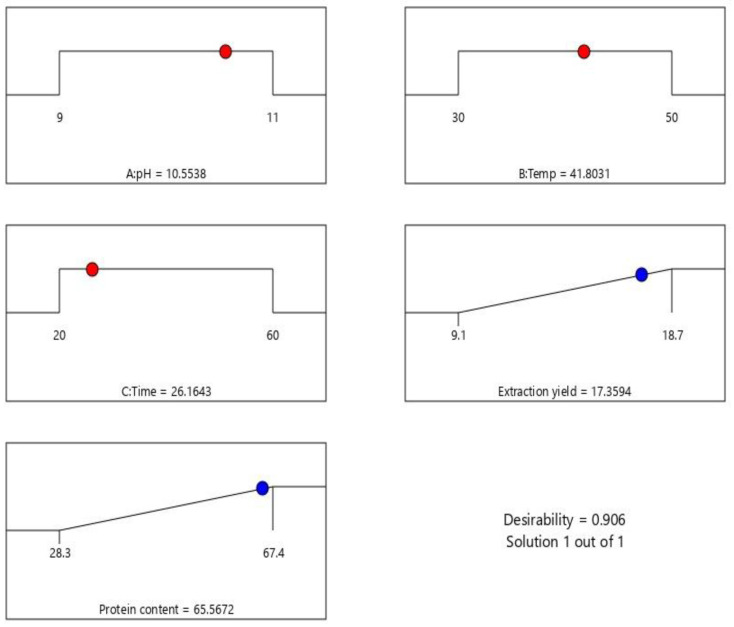
Optimized conditions of ultrasound-assisted alkaline extraction of protein from cherimoya seed.

Through all the data obtained in the experiments, it was possible to deduce the most optimal conditions for protein extraction from the cherimoya seeds (Figure 3). These conditions were established based on a compromise between obtaining the highest possible cherimoya-seed extraction yield and the highest possible protein content. Eventually, these conditions resulted in a pH of 10.55 (A), a temperature of 41.80 °C (B), and an extraction time of 26.16 min (C). With these conditions, the predicted extraction yield was calculated at 17.36%, and the concentrate’s protein content was 65.57%.

### 3.4. Thermal Properties of Cherimoya-Seed Proteins

Leaving aside the results of the optimization of the ultrasound-assisted extraction process, some of the techno-functional properties of the proteins obtained were tested. A comparison was made between the properties of the proteins obtained by the conventional method and those obtained by ultrasound extraction using optimal conditions. The properties of the proteins extracted were measured, reflecting the proteins’ ability to resist denaturation or aggregation as the temperature increased [27]. Thermogravimetric analysis (TGA) is a technique that is widely used in the study of the thermal properties of proteins, aiming to determine the best processing conditions for foodstuffs [28]. The results are represented on a graph showing two curves: the thermogravimetric curve (TGA curve) and the curve obtained by plotting the first derivative of the TGA curve (DTG curve) [29]. The former reflects the mass loss versus the temperature and the latter reports the temperature at which the degradation rate peaked.

**Figure 4:**
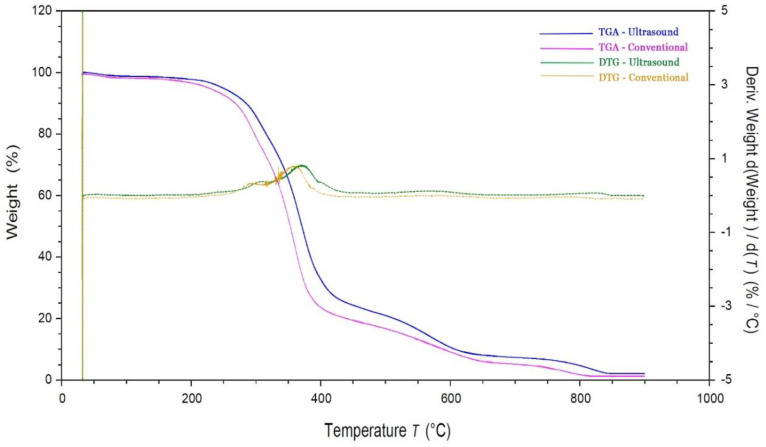
TGA and DTG thermograms of cherimoya-seed-protein concentrates obtained by ultrasound and conventional extraction techniques.

Figure 4 shows the degradation of the cherimoya-seed protein obtained by ultrasound and conventional extraction, which took place in different steps. Generally, the first mass loss was due to physical-chemical changes, such as the dehydration of the sample [30]. However, from 40 to 150 °C, the proteins extracted by ultrasound and conventional techniques showed no weight loss. This was because the samples were previously freeze-dried. The next step occurred at different temperature ranges, depending on the type of extraction used. Between 175 and 200 °C, the proteins extracted by the conventional method started to lose weight due to protein denaturation. In the case of the proteins extracted by ultrasound, this occurred between 225 and 300 °C, which showed that the proteins extracted by ultrasound were thermally more stable. These results are in agreement with [31,32], which indicate that proteins extracted using low sonication times are more susceptible to thermal treatments due to hydrophobic bond breaking. By contrast, those extracted by sonication for more than 20 min undergo a restoration of these hydrophobic bonds between protein molecules, thus increasing their thermal resistance.

As shown in the DTG curves, the maximum degradation rate of the protein obtained by ultrasound occurred at 375 °C, whereas for those extracted by the conventional method, it was at 360 °C. Finally, the third step was in the temperature range from 400 to 950 °C, at which point the curve slowed down, and the weight loss was due to char decomposition. Therefore, it can be concluded that proteins extracted by ultrasound have better thermal stability, which makes them a good option to be added to food products that require heat treatment, ensuring the maintenance of their functional properties [33,34].

### 3.5. Techno-Functional Properties of Cherimoya-Seed Proteins

The techno-functional properties, including water-holding capacity (WHC), oil-binding capacity (OBC), foam capacity (FC), and foam stability (FS), as well as the values for the physicochemical properties (extraction yield and protein content), are shown in Table 3. As mentioned in the previous section, the WHC is the ability of the polar groups of the protein to bind to water molecules and retain them for as long as possible. The OBC is the ability of the protein to bind to oils through its non-polar parts and retain these oils for as long as possible, FC is the ability of the protein to generate foams, and FS is the capacity of the protein to make these generated foams as stable as possible [35]. The WHC and OBC of the cherimoya-seed-protein concentrate obtained by ultrasound-assisted alkaline extraction were 3.9 (g water/g) and 4.11 (g oil/g), respectively. Regarding the values obtained for the techno-functional properties, a general trend of better values for all the properties was observed in the protein extracted using ultrasound, except of the FS, whose value was higher in the proteins obtained by the conventional method. These results agree with those of similar studies, which showed that ultrasound-assisted extractions can modify techno-functional properties [26,36].

## 4. Conclusions

Recent increases in the popularity of plant proteins are attributed to consumers’ increased knowledge of food components, their need for clean, lean protein, and their interest in natural and sustainable food sources. Numerous innovative technologies have been employed to enhance the extraction yield and reduce the duration of treatment and solvent consumption during alkaline extraction. These assistance techniques are especially necessary to extract intracellular proteins from the polysaccharide complexes in plant-cell walls. The pH at 10.5 and temperature of 41.8 °C for 26.1 min were considered the optimal ultrasound-assisted alkaline-extraction conditions, since they provided the maximum extraction yield (17.3%) and protein content (65.6%). The combination of the ultrasonication with the alkaline extraction resulted in a 6% higher extraction yield and 12% higher protein content in the cherimoya-seed protein compared to the classic alkaline technique. The ultrasound-assisted alkaline-extraction technique enhanced the cherimoya-seed protein’s thermal stability and functional properties.

## Figures and Tables

**Table 1:** Uncoded and coded values of the extraction-condition factors and observed responses.

	Factors			Responses	
Run	A: pH	B: Temperature °C	C: Time min	Extraction Yield %	Protein Content %
1	9 (−1)	50 (+1)	40 (0)	11.2	37.1
2	9 (−1)	40 (0)	20 (−1)	12.4	41.3
3	11 (+1)	40 (0)	20 (−1)	17.1	63.2
4	10 (0)	40 (0)	40 (0)	15.3	64.5
5	10 (0)	30 (−1)	60 (+1)	10.5	46.9
6	10 (0)	30 (−1)	20 (−1)	11.6	44.1
7	9 (−1)	30 (−1)	40 (0)	9.1	35.8
8	10 (0)	40 (0)	40 (0)	15.5	65.2
9	10 (0)	40 (0)	40 (0)	15	66.8
10	10 (0)	50 (+1)	60 (+1)	10.4	28.3
11	11 (+1)	40 (0)	60 (+1)	12.8	32.7
12	10 (0)	50 (+1)	20 (−1)	18.7	47.5
13	11 (+1)	30 (−1)	40 (0)	14.7	58.1
14	11 (+1)	50 (+1)	40 (0)	15.3	36.9
15	10 (0)	40 (0)	40 (0)	14.2	67.4
16	9 (−1)	40 (0)	60 (+1)	9.1	31.8

**Table 2:** Analysis of variance for the fitted second-order polynomial models.

	Extraction Yield			Protein Content		
Source	Coefficient Estimate	F-Value	*p*-Value	Coefficient Estimate	F-Value	*p*-Value
Model	15.00	20.15	0.0008	65.98	24.72	0.0005
A-pH	2.26	62.88	0.0002	5.61	19.40	0.0045
B-Temp	1.21	18.06	0.0054	−4.39	11.86	0.0137
C-Time	−2.13	55.47	0.0003	−7.05	30.61	0.0015
AB	−0.3750	0.8637	0.3886	−5.62	9.74	0.0205
AC	−0.2500	0.3839	0.5583	−5.25	8.49	0.0269
BC	−1.80	19.90	0.0043	−5.50	9.32	0.0224
A^2^	−1.19	8.66	0.0258	−11.72	42.34	0.0006
B^2^	−1.24	9.41	0.0220	−12.27	46.40	0.0005
C^2^	−0.9625	5.69	0.0544	−12.00	44.35	0.0006
Lack of Fit		2.99	0.1964		13.20	0.0310
R^2^	0.9680			0.9737		
Adj-R^2^	0.9299			0.9344		
C.V. (%)	6.06			7.51		

**Table 3:** Physicochemical and functional properties of cherimoya-seed-protein concentrate obtained by different extraction techniques.

	Extraction Technique	
Parameters	Conventional	Ultrasound-Assisted
Extraction yield (g/100 g)	12.2 ± 1.3 ^b^	18.3 ± 1.7 ^a^
Protein content (g/100 g)	54.5 ± 4.6 ^b^	66.1 ± 3.1 ^a^
Water-holding capacity (g water/g)	3.34 ± 0.31 ^b^	3.90 ± 0.17 ^a^
Oil-binding capacity (g oil/g)	3.37 ± 0.19 ^c^	4.11 ± 0.09 ^a^
Foam capacity (%)	165.8 ± 14.6 ^b^	192.5 ± 9.9 ^a^
Foam stability (%)	53.6 ± 5.9 ^a^	52.8 ± 4.5 ^a^

Different letters in each row indicate significant differences (*p* < 0.05).

## Data Availability

All data generated or analyzed during this study are included in this published article.
